# Uncovering Modern Clinical Applications of Fuzi and Fuzi-Based Formulas: A Nationwide Descriptive Study With Market Basket Analysis

**DOI:** 10.3389/fphar.2021.641530

**Published:** 2021-04-27

**Authors:** Chi-Jung Tai, Mohamed El-Shazly, Yi-Hong Tsai, Dezső Csupor, Judit Hohmann, Yang-Chang Wu, Tzyy-Guey Tseng, Fang-Rong Chang, Hui-Chun Wang

**Affiliations:** ^1^ Graduate Institute of Natural Products, College of Pharmacy, Kaohsiung Medical University, Kaohsiung, Taiwan; ^2^ Department of Family Medicine, Pingtung Hospital, Ministry of Health and Welfare, Pingtung, Taiwan; ^3^ Department of Pharmacognosy, Faculty of Pharmacy, Ain-Shams University, Organization of African Unity Street, Cairo, Egypt; ^4^ Department of Pharmaceutical Biology, Faculty of Pharmacy and Biotechnology, German University in Cairo, Cairo, Egypt; ^5^ Department of Pharmacognosy, Faculty of Pharmacy, University of Szeged, Szeged, Hungary; ^6^ Graduate Institute of Integrated Medicine, China Medical University, Taichung, Taiwan; ^7^ Chinese Medicine Research and Development Center, China Medical University Hospital, Taichung, Taiwan; ^8^ Department of Family Medicine, Kaohsiung Medical University Hospital, Kaohsiung Medical University, Kaohsiung, Taiwan; ^9^ Department of Marine Biotechnology and Resources, National Sun Yat-sen University, Kaohsiung, Taiwan; ^10^ Drug Development and Value Creation Research Center, Kaohsiung Medical University, Kaohsiung, Taiwan; ^11^ Department of Medical Research, Kaohsiung Medical University Hospital, Kaohsiung Medical University, Kaohsiung, Taiwan

**Keywords:** aconitum, clinical epidemiology, data mining, Fuzi, market basket analyses

## Abstract

**Background:** As time evolved, traditional Chinese medicine (TCM) became integrated into the global medical system as complementary treatments. Some essential TCM herbs started to play a limited role in clinical practices because of Western medication development. For example, Fuzi (Aconiti Lateralis Radix Praeparata) is a toxic but indispensable TCM herb. Fuzi was mainly used in poor circulation and life-threatening conditions by history records. However, with various Western medication options for treating critical conditions currently, how is Fuzi used clinically and its indications in modern TCM are unclear. This study aimed to evaluate Fuzi and Fuzi-based formulas in modern clinical practices using artificial intelligence and data mining methods.

**Methods:** This nationwide descriptive study with market basket analysis used a cohort selected from the Taiwan National Health Insurance database that contained one million national representatives between 2003 and 2010 used for our analysis. Descriptive statistics were performed to demonstrate the modern clinical indications of Fuzi. Market basket analysis was calculated by the Apriori algorithm to discover the association rules between Fuzi and other TCM herbs.

**Results:** A total of 104,281 patients using 405,837 prescriptions of Fuzi and Fuzi-based formulas were identified. TCM doctors were found to use Fuzi in pulmonary (21.5%), gastrointestinal (17.3%), and rheumatologic (11.0%) diseases, but not commonly in cardiovascular diseases (7.4%). Long-term users of Fuzi and Fuzi-based formulas often had the following comorbidities diagnosed by Western doctors: osteoarthritis (31.0%), peptic ulcers (29.5%), hypertension (19.9%), and COPD (19.7%). Patients also used concurrent medications such as H2-receptor antagonists, nonsteroidal anti-inflammatory drugs, β-blockers, calcium channel blockers, and aspirin. Through market basket analysis, for the first time, we noticed many practical Fuzi-related herbal pairs such as Fuzi–Hsihsin (Asari Radix et Rhizoma)–Dahuang (Rhei Radix et Rhizoma) for neurologic diseases and headache.

**Conclusion:** For the first time, big data analysis was applied to uncover the modern clinical indications of Fuzi in addition to traditional use. We provided necessary evidence on the scientific use of Fuzi in current TCM practices, and the Fuzi-related herbal pairs discovered in this study are helpful to the development of new botanical drugs.

## Introduction

Traditional and complementary medicine (T&CM) has played an increasingly significant role in the global medical system. The World Health Organization (WHO) started to facilitate the integration of T&CM into national health systems in member states, produced guidelines for T&CM complying with international standards, stimulated strategic research into T&CM, and advocated the rational use of T&CM through clinical evidence (World Health Organization, 2013). Traditional Chinese Medicine (TCM) is the most popular T&CM system worldwide, especially in Asia and North America. Most Western doctors and researchers come to know the clinical indications of TCM herbs from TCM books such as Shen Nong Ben Cao Jing and Shang Han Lun (Tai et al., 2015). However, modern clinical indications of TCM herbs might differ from those of ancient times because the current TCM doctors approach patients with innovative strategies using TCM herbs in combination with Western medications (Chen et al., 2015).

For example, Fuzi (Aconiti Lateralis Radix Praeparata) has been widely used in TCM for more than 2000 years (Nyirimigabo et al., 2015). In TCM, Fuzi and Fuzi-based formulas were prescribed to improve circulation in patients with cold extremities, weak pulse, general weakness, and fatigue (Singhuber et al., 2009). The applications of Fuzi were advocated in Shang Han Lun, which was an essential TCM book written by Zhon-Ging Zhang in the Eastern Han Dynasty around 150–209 AD. Zhang introduced some Fuzi-based formulas, including Sini Tang (Aconiti Lateralis Radix Praeparata, Zingiberis Rhizoma, and Glycyrrhizae Radix et Rhizoma) and Jenwu Tang (Aconiti Lateralis Radix Praeparata, Poria, Paeoniae Rubra Radix, Zingiberis Rhizoma, and Atractylodis Macrocephalae Rhizoma) (Zhang et al., 1999). After then, these famous Fuzi-based formulas have been used clinically, especially for the critical condition, in TCM for thousands of years. The use of Fuzi was also advocated by modern TCM doctors such as Hai-Ha Ni in the United States and Bu-Tao Chang in Taiwan (Tai et al., 2015).

Fuzi was also used in Ayurvedic and Kampo medicine to treat several clinical conditions such as neuralgia, polyuria, poor circulation, heart failure (HF), and even impotence (Tai et al., 2015). In Japan, some famous Fuzi-based formulas were directly adopted by Kampo medicine. For example, Shinbuto resembled Jenwu Tang and Shigyakuto was derived from Sini Tang. In addition, Kampo medicine doctors introduced some innovative Fuzi-based commercial formulas for new clinical indications, such as Gosha-jinki-gan (Aconiti Lateralis Radix Praeparata, Rehmanniae Radix, Achyranthis Bidentatae Radix, Corni Sarcocarpium, Dioscoreae Rhizoma, Plantaginis Semen, Alisma Rhizoma, Poria, Moutan Radicis Cortex, and Cinnamomi Cortex) for lymphedema (Abe, 2002). These applications supported the importance of Fuzi, which was not only limited to TCM, in modern medical system.

Although the therapeutic effect of Fuzi and Fuzi-based formula are widely recognized among TCM doctors and therapists, the toxicity of Fuzi has always been the main concern for their applications and further promotion (Lu et al., 2010). The major pharmacologic components of Fuzi are diester-diterpenoid alkaloids (DDAs, including aconitine, mesaconitine, and hypaconitine) and monoester-diterpenoid alkaloids (MDAs, including benzoylaconine, benzoylmesaconine, and benzoylhypaconine), which might provoke cardiac arrhythmias (Csupor et al., 2009). More than 600 cases of aconitum poisoning were published worldwide, mostly in China and Hong Kong (Chan, 2002). Furthermore, even in Asia, Western medicine has significantly replaced TCM in the treatment of critical conditions in recent years. Therefore, it is not known whether the clinical indications of Fuzi have changed over time.

In Taiwan, TCM has been formally adopted into the Taiwan National Health Insurance (NHI) system since 1996 (Chu et al., 2015), and the system reimburses more than 95% of TCM services. Given that one-third of the Taiwanese population uses TCM (Huang C. W. et al., 2018), the Taiwan NHI database is a unique engine to generate evidence-based results to improve practices, policy-making, and TCM integrated with Western medicine.

Analyzing the Taiwan NHI database, previous epidemiologic TCM studies have successfully observed the effectiveness and adverse effects of TCM formulas on a specific single disease (Lai et al., 2013; Chang et al., 2016). At present, there is no study using a well-documented database to explore the modern clinical applications of single TCM herbs or well-known formulas, or to expand their clinical indications for treating diseases or syndromes. To reveal the essence and art of the TCM, we aimed to demonstrate the use of artificial intelligence (AI) and data mining methods to shed new light on TCM use in Western medicine predominant populations. Herein, the Fuzi and Fuzi-based formulas are as representatives.

## Methods

### Data Source

This retrospective cohort study using a longitudinal cohort of Taiwan NHI database comprises a randomly sampled representative database of one million people from all NHI enrollees alive in 2000 (National Health Insurance Administration, 2014). The single-payer NHI program has enrolled more than 99% of the 23 million people in Taiwan's population, which was launched in Taiwan in 1995 (Huang et al., 2016). The Taiwan NHI database contains the beneficiaries’ medical records, such as demographic data, inpatient records, outpatient prescriptions, and expenditure for healthcare services. The Taiwan NHI database is a de-linked and anonymized database, which well protects the privacy of patient information. TCM products processed by standard pharmacopeial methods were commonly prescribed in the healthcare system in Taiwan. Moreover, TCM doctors in Taiwan began to use the International Classification of Diseases Revision, Ninth Revision, Clinical Modification (ICD-9-CM) codes for their clinical diagnosis since May 2002. Therefore, we used a subset of the Taiwan NHI database from 2003 to 2010 for further analysis. This study was approved by the Institutional Review Board of Antai Medical Care Cooperation Antai-Tian-Sheng Memorial Hospital (protocol number: 18-015-C; approval date: Feb. 21st, 2018).

### Fuzi and Fuzi-Based Formulas

We searched for prescriptions of Fuzi and Fuzi-based formulas from outpatient medical claims with the specific drug codes in the cohort between January 2003 and December 2010 (Supplementary Table S1). There were four famous Fuzi-based formulas, including Sini Tang, Jenwu Tang, Mahuang-Fuzi-Hsihsin Tang (Ephedra Herba, Aconiti Lateralis Radix Praeparata, and Asari Radix et Rhizoma), and Fuzi-Lizhong Tang (Aconiti Lateralis Radix Praeparata, Ginseng Radix, Zingiberis Rhizoma, Atractylodis Macrocephalae Rhizoma, and Glycyrrhizae Radix et Rhizoma), commercially available in the Taiwan NHI system. Other commercially unavailable Fuzi-based formulas have not been recorded in the Taiwan NHI system.

### Descriptive Study Design

First, the prescription numbers of Fuzi and Fuzi-based formulas and their growth rate were estimated. In Taiwan, only Western medicine doctor can prescribe Western medications. Similarly, only TCM doctors can prescribe TCM drugs. To specify the primary diagnosis documented by the TCM doctors prescribing Fuzi, we grouped the primary ICD-9-CM diagnosis as diagnostic categories for analysis (Supplementary Table S2). The diagnostic categories were divided into 19 conditions, including headache, cancer, infection, benign tumor, unspecific complaints, as well as neurological, psychological, rheumatological, urological, obstetrical/gynecological, hematological, dermatological, cardiovascular, endocrine, pulmonary, gastrointestinal, musculoskeletal, sensory organs, and male reproductive systems disorders.

Second, the third quartile patients who experienced higher prescription numbers or prescription duration were defined as the long-term users of Fuzi or Fuzi-based formulas. To identify long-term users' underlying comorbidities diagnosed by Western medicine doctors, we analyzed the selected corresponding ICD-9-CM codes in outpatient medical claims before the prescription date of Fuzi or Fuzi-based formulas (Supplementary Table S3). The medical comorbidities included hypertension, DM, cerebrovascular disease, HF, ischemic heart disease, cardiac arrhythmias, hyperlipidemia, chronic obstructive pulmonary disease (COPD), chronic kidney disease (CKD), chronic liver disease, esophageal disease, peptic ulcers, thyroid disease, rheumatoid arthritis (RA), ankylosing spondylitis (AS), systemic lupus erythematosus (SLE), osteoarthritis, and cancer.

Finally, the common Western medications were evaluated, including aspirin, clopidogrel, lipid-lowering agents (statins, fibrates, and other lipid-lowering agents), xanthine oxidase inhibitors, uricosuric agents, colchicine, β-blockers, calcium channel blockers (CCBs), angiotensin-converting enzyme inhibitors (ACEI)/angiotensin receptor blockers (ARBs), H2-receptor antagonists, proton pump inhibitors (PPIs), nonsteroidal anti-inflammatory drugs (NSAIDs), metformin, sulfonylurea, insulin, COPD inhalants, thyroxine, and α-adrenoreceptor antagonists (Supplementary Table S4). To identify the co-use of Western medications with Fuzi, we identified the mentioned Western medications by the corresponding anatomical therapeutic chemical (ATC) codes.

### Market Basket Analysis

Market basket analysis, a data mining technique introduced by Agrawal et al., is frequently used to discover the rules of association between sets of items in large databases (Agrawal et al., 1993). In our study, 442 kinds of TCM single herbs used in the Taiwan NHI system were evaluated by the market basket analysis to reveal their combination principle of Fuzi, so-called Fuzi-related pairs. The mentioned TCM herbal drugs with significant findings, including their Latin names and scientific names, were listed in Supplementary Table S5.

The association rules derived from market basket analysis were demonstrated in the following three measures: support, confidence, and lift (Tan et al., 2005). 1) The support was the relative frequency that the rule showed up in the given dataset. 2) The confidence measured the reliability of the inference made by a rule. For example, an association rule X (antecedent factor) to Y (consequent factor) was established by the estimate of the conditional probability of Y given X (antecedent confidence) or X given Y (consequent confidence), and high confidence indicated a strong association rule. This study generated frequent TCM pairs that have the support of at least 5%. 3) The lift was the likelihood of prescribing both drugs compared with a single drug. Lift value above one means there was a positive correlation between the two drugs. The higher the lift value, the more likely the two drugs were to be prescribed together.

### Statistical Analyses

Demographic statistics were demonstrated as means ± standard deviation, median, interquartile, or number (percentages). SAS version 9.4 (SAS Institute Inc, Cary, NC, United States) was used for data collection, and statistical analyses. Market basket analysis was calculated by the Apriori algorithm using Python version 3.7.

## Results

### Clinical Demographics and the Prescriptions of Fuzi and Fuzi-Based Formulas

A total of 104,281 patients using 405,837 prescriptions of Fuzi and Fuzi-based formulas were identified between January 2003 and December 2010. Among the users, 11.7% were older adults (≥65 years old), 9.5% were children or adolescents (≤18 years old). The mean age of the users was 41.2 ± 18.0 years. The users received 1.4 ± 0.7 types of Fuzi prescriptions during the evaluation period, and the average prescription number in each patient was 3.9 ± 7.2 (median, 2; third quartile, 4). The average duration of the prescriptions was 304.3 ± 589.0 days (median, 8; third quartile, 286). The number of long-term users (number of prescriptions >4) was 28,978 patients, including 12.5% older adults and 8.3% of children or adolescents.

**FIGURE 1:**
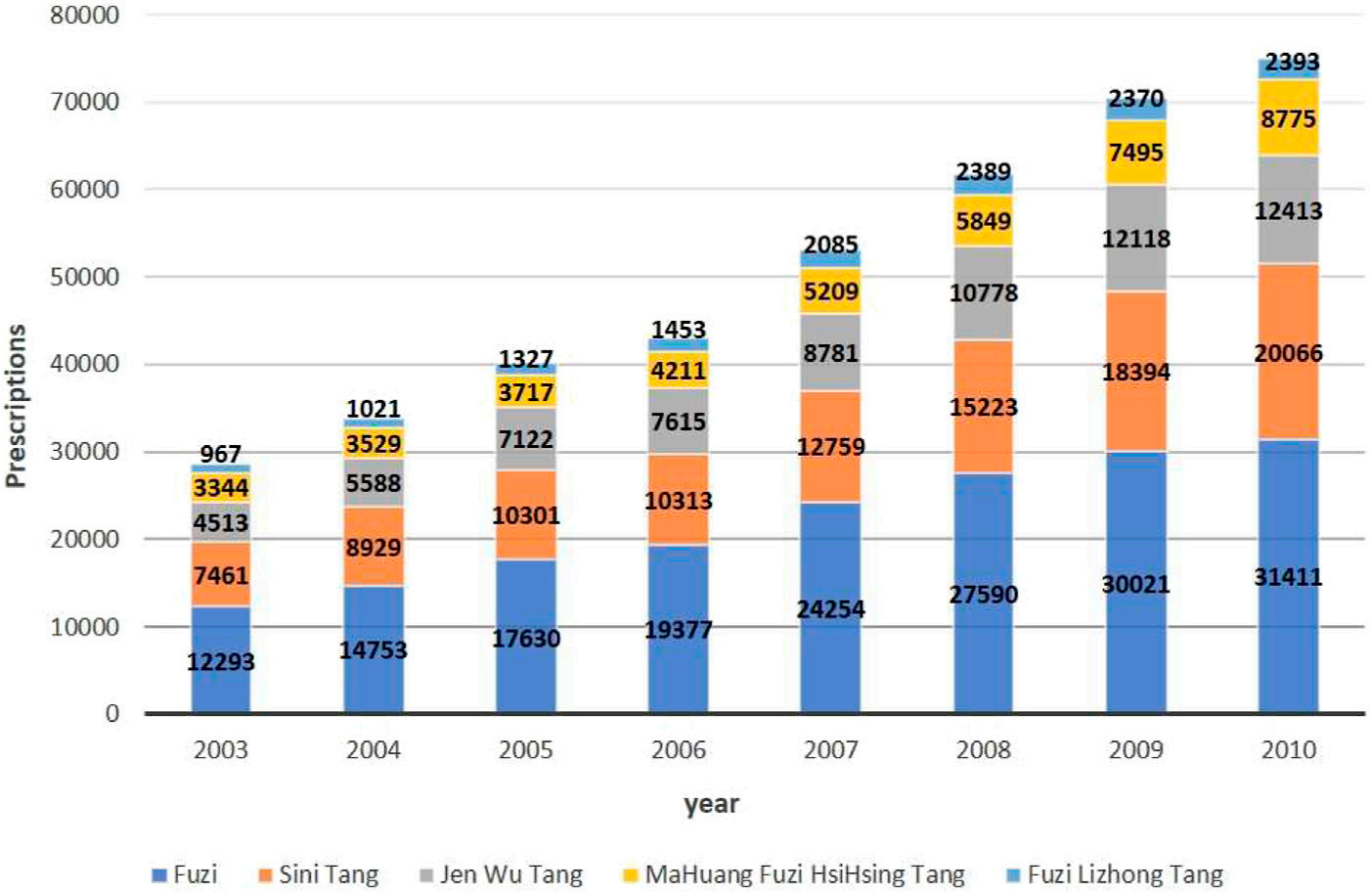
National trends in the prescriptions of Fuzi and Fuzi-based formulas during 2003–2010.

The total prescriptions of Fuzi and Fuzi-based formulas increased annually, from 28,578 prescriptions in 2003 to 75,058 prescriptions in 2010 (Figure 1). Among the prescriptions, 43.7% were Fuzi as a single component, and the others were Fuzi-based formulas. A small fraction of prescriptions (2,324, 1.3%) used Fuzi in addition to the Fuzi-based formulas. According to the number of prescriptions, the prescriptions of Fuzi-based formula from high to low were Sini Tang, Jenwu Tang, Mahuang-Fuzi-Hsihsin Tang, and Fuzi-Lizhong Tang. The compound annual growth rate of prescriptions of Fuzi, Sini Tang, Jenwu Tang, Mahuang-Fuzi-Hsihsin Tang, and Fuzi-Lizhong Tang was 14.3, 15.2, 15.6, 14.8, and 13.8%, respectively.

**FIGURE 2:**
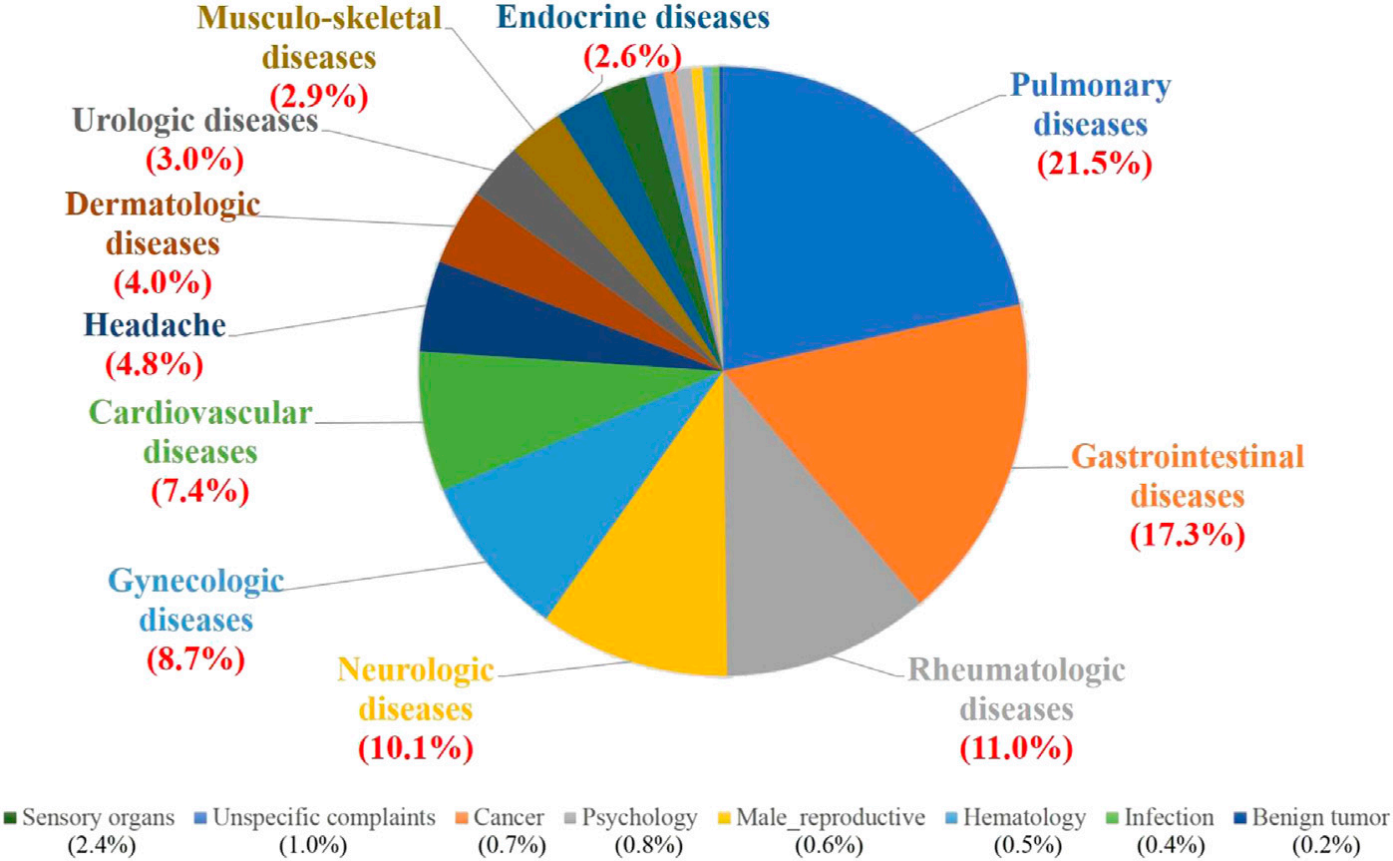
Clinical indications of prescriptions of Fuzi and Fuzi-based formulas from TCM doctors in systematic categories. The percentage of total Fuzi-related prescription numbers for indicated diseases is shown in parentheses.

Of the total number of prescriptions, Fuzi and Fuzi-based formulas were commonly used in clinical conditions including pulmonary (21.5%), gastrointestinal (17.3%), rheumatologic (11.0%), neurologic (10.1%), and gynecologic (8.7%) diseases (Figure 2). Of surprise, Fuzi and Fuzi-based formulas were not commonly used in cardiovascular diseases (7.4%) and cancer complementary treatment (0.7%). The details in the prescriptions of Fuzi and Fuzi-based formulas were listed in Table 1.

**TABLE 1:** Clinical indications of prescriptions of Fuzi and Fuzi-based formulas from TCM doctors in systematic categories.

Category	Fuzi*n* = 179,653, n (%)	Sini Tang*n* = 104,674, n (%)	Jenwu Tang*n* = 69,864, n (%)	Mahuang-Fuzi-Hsihsin Tang	Fuzi-Lizhong Tang*n* = 14,090, n (%)
				*n* = 42,592, n (%)	
Gastrointestinal system	35,336 (19.7)	17,217 (16.4)	8,889 (12.7)	3,263 (7.7%	6,414 (45.5)
Pulmonary system	32,119 (17.9)	14,278 (13.6)	14,539 (20.8)	25,876 (60.8)	1,468 (10.4)
Rheumatology	21,527 (12.0)	15,553 (14.9)	4,299 (6.2)	3,018 (7.1)	812 (5.8)
Neurology	17,470 (9.7)	12,695 (12.1)	8,613 (12.3)	1771 (4.2)	879 (6.2%)
Gynecology	15,739 (8.8)	12,738 (12.2)	4,212 (6.0)	1,607 (3.8)	1,563 (11.1)
Cardiovascular system	13,585 (7.6)	7,941 (7.6)	7,685 (11.0)	762 (1.8)	541 (3.8)
Headache	8,359 (4.7)	4,420 (4.2)	4,427 (6.3)	2,357 (5.5)	307 (2.2)
Dermatology	6,767 (3.8)	4,267 (4.1)	4,186 (6.0)	988 (2.3)	423 (3.0)
Urology	5,724 (3.2)	2,324 (2.2)	3,689 (5.3)	439 (1.0)	354 (2.5)
Endocrine system	5,499 (3.1)	2,797 (2.7)	1,692 (2.4)	466 (1.1)	267 (1.9)
Musculoskeletal system	4,928 (2.7)	4,211 (4.0)	1,419 (2.0)	1,010 (2.4)	314 (2.2)
Sensory organs	3,944 (2.2)	1960 (1.9)	3,446 (4.9)	414 (1.0)	211 (1.5)
Unspecific complaints	1949 (1.1)	1,208 (1.2)	552 (0.8)	155 (0.4)	116 (0.8)
Cancer	1,562 (0.9)	597 (0.6)	434 (0.6)	106 (0.2)	13 (0.1)
Psychology	1704 (0.9)	730 (0.7)	578 (0.8)	118 (0.3)	102 (0.7)
Male reproductive system	1,289 (0.7)	410 (0.4)	664 (1.0)	91 (0.2)	37 (0.3%)
Hematology	883 (0.5)	671 (0.6)	326 (0.5)	45 (0.1)	57 (0.4)
Infectious diseases	843 (0.5)	420 (0.4)	130 (0.2)	62 (0.1)	182 (1.3)
Benign tumors	426 (0.2)	237 (0.2)	84 (0.1)	44 (0.1)	30 (0.2)

Corresponding ICD-9-CM codes of each category were listed in Supplementary Table S2. The sensory system includes ophthalmology and otolaryngology.

### Clinical Comorbidities and Related Western Medications

From the viewpoint of Western medicine, the most frequent comorbidities of long-term Fuzi or Fuzi-based formulas users were osteoarthritis (31.0%), gastric or peptic ulcers (29.5%), hypertension (19.9%), COPD (19.7%), hyperlipidemia (16.4%), and chronic liver diseases (16.3%) (Table 2). The users of Jenwu Tang experienced the highest prevalence of osteoarthritis (38.4%), COPD (24.6%), hypertension (26.1%), DM (13.7%), and cerebrovascular disease (9.4%) among all users of Fuzi-based formulas (Table 2). The users of Fuzi and Fuzi-based formulas often took Western medications such as NSAIDs (67.8%), H2-receptor antagonists (29.9%), β-blockers (23.9%), CCBs (21.8%), ACEI/ARBs (16.3%), and aspirin (14.5%) (Table 3). These results showed that long-term Fuzi users suffered from some underlying comorbidities and took related Western medications.

**TABLE 2:** Underlying comorbidities diagnosed by Western medicine doctors of patients who long-term used fuzi and fuzi-based formulas.

Comorbidities	Fuzi and Fuzi-based formulas*n* = 28,978, n (%)	Fuzi*n* = 20,233, n (%)	Sini Tang*n* = 15,286, n (%)	Jenwu Tang*n* = 11,177, n (%)	Mahuang-Fuzi-Hsihsin Tang*n* = 7,549, n (%)	Fuzi-Lizhong Tang*n* = 2,820, n (%)
Osteoarthritis	8,971 (31.0)	6,867 (33.9)	5,253 (34.4)	4,291 (38.4)	2,126 (28.2)	968 (34.3)
Peptic ulcers	8,543 (29.5)	6,755 (33.4)	5,108 (33.4)	4,119 (36.9)	2,379 (31.5)	1,173 (41.6)
Hypertension	5,768 (19.9)	4,242 (21.0)	3,006 (19.7)	2,912 (26.1)	1,156 (15.3)	580 (20.6)
COPD	5,695 (19.7)	4,269 (21.1)	3,099 (20.3)	2,754 (24.6)	1792 (23.7%)	625 (22.2%)
Hyperlipidemia	4,740 (16.4)	3,635 (18.0)	2,737 (17.9)	2,428 (21.7)	1,088 (14.4)	496 (17.6)
Chronic liver disease	4,717 (16.3)	3,641 (18.0)	2,773 (18.1)	2,229 (19.9)	1,203 (15.9)	572 (20.3)
Diabetes mellitus	3,002 (10.4)	2,260 (11.2)	1741 (11.4)	1,527 (13.7)	642 (8.5)	299 (10.6)
Esophageal disease	2,577 (8.9)	2084 (10.3)	1,666 (10.9)	1,435 (12.8)	758 (10.0)	373 (13.2)
Thyroid disease	2,194 (7.6)	1776 (8.8)	1,396 (9.1)	1,093 (9.8)	591 (7.8	296 (10.5)
RA, SLE, or AS	1901 (6.6)	1,529 (7.6)	1,221 (8.0)	982 (8.8)	539 (7.1)	333 (8.3)
Cerebrovascular disease	1871 (6.5)	1,385 (6.8)	1,041 (6.8)	1,054 (9.4)	383 (5.1)	168 (6.0)
Cardiac arrhythmias	1727 (6.0)	1,389 (6.9)	1,057 (6.9)	1,006 (9.0)	515 (6.8)	239 (8.5)
Ischemic heart disease	1,505 (5.2)	1,195 (5.9)	893 (5.8)	863 (7.7)	343 (4.5)	163 (5.8)
Cancer	1,181 (4.1)	915 (4.5)	662 (4.3)	586 (5.2)	270 (3.6)	128 (4.5)
Heart failure	744 (2.6)	576 (2.8)	421 (2.8)	458 (4.1)	159 (2.1)	72 (2.6)
Chronic kidney disease	449 (1.6)	324 (1.6)	207 (1.4)	280 (2.5)	77 (1.0)	57 (2.0)

Corresponding ICD-9-CM codes of each disease were listed in Supplementary Table S3. AS = ankylosing spondylitis; COPD = chronic obstructive pulmonary disease; RA = rheumatoid arthritis; SLE = systemic lupus erythematosus.

**TABLE 3:** Common Western medications prescribed by Western medicine doctors in patients who long-term use Fuzi and Fuzi-based formulas.

Western medications	Fuzi and Fuzi-based formulas*n* = 28,978, n (%)	Fuzi*n* = 20,233, n (%)	Sini Tang*n* = 15,286, n (%)	Jenwu Tang*n* = 11,177, n (%)	Mahuang-Fuzi-Hsihsin Tang*n* = 7,549, n (%)	Fuzi-Lizhong Tang*n* = 2,820, n (%)
NSAIDs	19,648 (67.8)	14,545 (71.9)	11,522 (75.4)	8,516 (76.6)	5,767 (76.4)	2,184 (77.4)
H_2_-receptor antagonist	8,663 (29.9)	6,669 (33.0)	5,523 (36.1)	4,070 (36.6)	2,642 (35.0)	1,134 (40.2)
*β*-Blockers	6,930 (23.9)	5,306 (26.2)	4,201 (27.5)	3,529 (31.7)	1788 (23.7)	786 (27.9)
CCBs	6,306 (21.8)	4,810 (23.8)	3,692 (24.2)	3,264 (29.4)	1,538 (20.4)	697 (24.7)
ACEI/ARB	4,732 (16.3)	3,603 (17.8)	2,789 (18.2)	2,485 (22.4)	1,161 (15.4)	523 (18.5)
Aspirin	4,204 (14.5)	3,235 (16.0)	2,553 (16.7)	2,169 (19.5)	1,038 (13.8)	452 (16.0)
Lipid-lowering agents	3,699 (12.8)	2,798 (13.8)	2,235 (14.6)	1881 (16.9)	915 (12.1)	401 (14.2)
Sulfonylurea	3,162 (10.9)	2,401 (11.9)	1953 (12.8)	1,608 (14.5)	829 (11.0)	359 (12.7)
Metformin	3,076 (10.6)	2,334 (11.5)	1921 (12.6)	1,574 (14.2)	788 (10.4)	367 (13.0)
Proton pump inhibitor	2,354 (8.1)	1858 (9.2)	1,564 (10.2)	1,234 (11.1)	680 (9.0)	316 (11.2)
α-Adrenoreceptor antagonists	1855 (6.4)	1,417 (7.0)	1,124 (7.4)	968 (8.7)	465 (6.2)	209 (7.4)
COPD inhalants	1,637 (5.6)	1,255 (6.2)	940 (6.1)	864 (7.8)	571 (7.6)	168 (6.0)
Colchicine	1,315 (4.5)	1,019 (5.0)	819 (5.4)	649 (5.8)	346 (4.6)	138 (4.9)
Uricosuric agents	1,141 (3.9)	896 (4.4)	701 (4.6)	597 (5.4)	296 (3.9)	126 (4.5)
XOIs	814 (2.8)	626 (3.1)	496 (3.2)	406 (3.7)	197 (2.6)	97 (3.4)
Insulin	675 (2.3)	514 (2.5)	393 (2.6)	399 (3.6)	159 (2.1)	77 (2.7)
Thyroxine	615 (2.1)	484 (2.4)	396 (2.6)	328 (3.0)	161 (2.1)	70 (2.5)
Clopidogrel	467 (1.6)	366 (1.8)	278 (1.8)	253 (2.3)	96 (1.3)	57 (2.0)

Corresponding ATC codes of each medication were listed in Supplementary Table S4. ACEI = angiotensin-converting enzyme inhibitors; ARB = angiotensin receptor blockers; CCBs = calcium channel blockers; NSAIDs = nonsteroidal anti-inflammatory drugs; XOIs = xanthine oxidase inhibitors.

### Fuzi-Related Herbal Pairs

Fuzi may be combined with other TCM herbs according to the knowledge and experience of the TCM doctors. We analyzed the co-prescription TCM single herbs with Fuzi. Of total Fuzi prescriptions, the top 10 Fuzi-related herbal pairs were Fuzi–Dahuang (Rhei Radix et Rhizoma) (16.5%), Fuzi–Hsihsin (Asari Radix et Rhizoma) (15.9%), Fuzi–Ganjiang (Zingiberis Rhizoma) (15.5%), Fuzi–Fuling (Poria) (13.8%), Fuzi–Baizhu (Atractylodis Macrocephalae Rhizoma) (10.3%), Fuzi–Quizhi (Cinnamomi Ramulus) (10.1%), Fuzi–Huangqi (Astragali Radix) (9.0%), Fuzi–Banxia (Pinelliae Rhizoma) (8.3%), Fuzi–Rouqui (Cinnamomi Cortex) (8.0%), and Fuzi-Gancao (Glycyrrhizae Radix et Rhizoma) (7.1%) (The scientific names of these herbs were listed in Supplementary Table S5). The rank of the top 10 TCM herbs prescribed in combination with Fuzi was altered in the classified subgroups of specific clinical conditions. Herein, we identified Fuzi-related single herbal pairs in six specific clinical conditions (Table 4).

**TABLE 4:** Top 10 Fuzi–single TCM herbal pairs prescribed by TCM doctors to treat patients with six specific clinical conditions.

1. Pulmonary diseases, total prescriptions n = 29,621, n (%)									
Hsihsin	Ganjiang	Mahuang	Dahuang	Quizhi	Fuling	Jiegeng	Gancao	Banxia	Huangqi
8,165 (27.6%)	5,012 (16.9%)	4,347 (14.7%)	3,230 (10.9%)	3,077 (10.4%)	3,072 (10.4%)	2,750 (9.3%)	2,567 (8.7%)	2,553 (8.6%)	2,465 (8.3%)

2. Gastrointestinal diseases, total prescriptions n = 33,013, n (%)									
Dahuang	Hsihsin	Fuling	Ganjiang	Banxia	Baizhu	Sharen	Quizhi	Rouqui	Huangqi
9,581 (29.0%)	6,861 (20.8%)	6,375 (19.3%)	5,616 (17.0%)	5,204 (15.8%)	3,201 (9.7%)	3,102 (9.4%)	2,480 (7.5%)	2,458 (7.4%)	2,414 (7.3%)

3. Rheumatological diseases, total prescriptions n = 19,265, n (%)									
Quizhi	Baizhu	Fuling	Ganjiang	Yanhusuo	Chuan-niuxi	Rouqui	Niuxi	Dahuang	Duzhong
3,317 (17.2%)	3,112 (16.2%)	2,648 (13.7%)	2,252 (11.7%)	2,215 (11.5%)	1835 (9.5%)	1810 (9.4%)	1835 (9.5%)	1,690 (8.8%)	1,613 (8.4%)

4. Neurologic diseases, total prescriptions n = 16,318, n (%)									
Ganjiang	Dahuang	Fuling	Hsihsin	Baizhu	Huangqi	Rouqui	Gancao	Quizhi	Suanzaoren
2,864 (17.6%)	2,433 (14.9%)	2,179 (13.4%)	1822 (11.2%)	1,545 (9.5%)	1,515 (9.3%)	1,435 (8.8%)	1,414 (8.7%)	1,390 (8.5%)	1,357 (8.3%)(8.3%)

5. Cardiovascular diseases, total prescriptions n = 12,387, n (%)									
Ganjiang	Baizhu	Dahuang	Fuling	Quizhi	Danshen	Huangqi	Rouqui	Hsihsin	Dangqui
1915 (15.5%)	1,543 (12.5%)	1,465 (11.8%)	1,427 (11.5%)	1,352 (10.9%)	1,299 (10.5%)	1,283 (10.4%)	1,114 (9.0%)	1,100 (8.9%)	903 (7.3%)

6. Headache, total prescription n = 12,063, n (%)									
Hsihsin	Dahuang	Ganjiang	Fuling	Quizhi	Baizhu	Yanhusuo	Chuanxiong	Gancao	Gegen
1980 (16.4%)	1707 (14.2%)	1,652 (13.7%)	1,630 (13.5%)	1,415 (11.7%)	1,353 (11.2%)	1,166 (9.7%)	1,090 (9.0%)	1,000 (8.3%)	933 (7.7%)

TCM, traditional Chinese medicine. The detailed identifications of the mentioned TCM herbal drugs, including Latin names and scientific names, are listed in supplementary Table S5.

**FIGURE 3:**
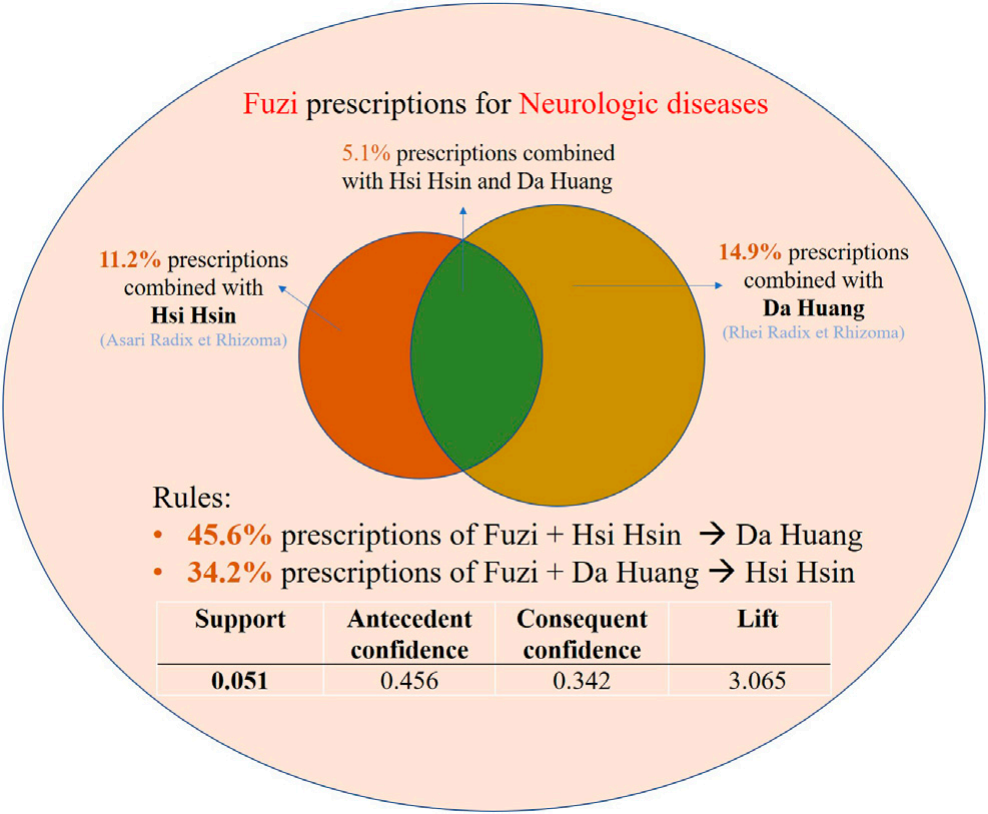
The interpretation example of the association rules of Fuzi–Dahuang–Hsihsin herbal pair derived from market basket analysis. The association rules were demonstrated in the following three measures: support, confidence, and lift. 1) The “support” was the relative frequency that the rule showed up in the given dataset. 2) The “confidence” measured the reliability of the inference made by a rule. For example, an association rule X (antecedent factor) to Y (consequent factor) was established by the estimate of the conditional probability of Y given X (antecedent confidence) or X given Y (consequent confidence), and high confidence indicated a strong association rule. 3) The “lift” was the likelihood of prescribing both drugs compared with a single drug. Lift value above one means there was a positive correlation between the two drugs. The higher the lift value, the more likely the two drugs were to be prescribed together.

In addition to Fuzi and single TCM herbal pairs, we conducted market basket analysis to find the association rules among Fuzi and multiple TCM herbs. For example, Fuzi–Hsihsin–Dahuang was a common TCM pair with the support of 5.1% (antecedent confidence, 0.456; consequent confidence, 0.342; Lift, 3.065) in neurologic diseases (Table 5). The association rules could be interpreted that 5.1% of Fuzi prescriptions for neurologic diseases, TCM doctors combined Hsihsin and Dahuang (Figure 3). Also, 45.6% of prescriptions comprising Fuzi and Hsihsin included Dahuang (Table 5 and Figure 3). 34.2% of prescriptions comprising Fuzi and Dahuang also combined with Hsihsin (Table 5 and Figure 3). The Fuzi–Hsihsin–Dahuang combination was also used in gastrointestinal diseases and headache (Table 5). Besides, the Fuzi–Fuling–Ganjiang was another common herbal pair used in neurologic diseases with the support of 5.4% (antecedent confidence, 0.306; consequent confidence, 0.403; Lift, 2.304).

**TABLE 5:** The combination principle of Fuzi and multiple TCM herbs discovered by market basket analysis for six clinical indications.

TCM pairs	Antecedent drug	Consequent Drug	Support	Antecedent confidence	Consequent confidence	Lift
**1. Pulmonary diseases**						
Fuzi–Hsihsin–Mahuang	Hsihsin	Mahuang	0.096	0.349	0.655	2.378
**2. Gastrointestinal diseases**						
Fuzi–Banxia–Dahuang	Banxia	Dahuang	0.103	0.653	0.355	2.254
Fuzi–Fuling–Dahuang	Fuling	Dahuang	0.091	0.470	0.313	1.623
Fuzi–Hsihsin–Dahuang	Hsihsin	Dahuang	0.143	0.689	0.494	2.378
Fuzi–Banxia–Fuling	Banxia	Fuling	0.093	0.590	0.482	3.057
Fuzi–Banxia–Hsihsin	Banxia	Hsihsin	0.097	0.614	0.466	2.955
Fuzi–Hsihsin–Fuling	Hsihsin	Fuling	0.076	0.364	0.392	1.889
**3. Rheumatological diseases**						
Fuzi–Fuling–Baizhu	Fuling	Baizhu	0.093	0.585	0.687	4.309
**4. Neurologic diseases**						
Fuzi–Hsihsin–Dahuang	Hsihsin	Dahuang	0.051	0.456	0.342	3.065
Fuzi–Fuling–Ganjiang	Fuling	Ganjiang	0.054	0.306	0.403	2.304
**5. Cardiovascular diseases**						
Fuzi–Fuling–Baizhu	Fuling	Baizhu	0.054	0.432	0.467	3.749
**6. Headache**						
Fuzi–Hsihsin–Dahuang	Hsihsin	Dahuang	0.054	0.331	0.391	2.398
Fuzi–Fuling–Baizhu	Fuling	Baizhu	0.063	0.563	0.468	4.205

The association rules were demonstrated in the following three measures: support, confidence, and lift. 1) The “support” was the relative frequency that the rule showed up in the given dataset. 2) The “confidence” measured the reliability of the inference made by a rule. For example, an association rule X (antecedent factor) to Y (consequent factor) was established by the estimate of the conditional probability of Y given X (antecedent confidence) or X given Y (consequent confidence), and high confidence indicated a strong association rule. 3) The “lift” was the likelihood of prescribing both drugs compared with a single drug. Lift value above one means there was a positive correlation between the two drugs. The higher the lift value, the more likely the two drugs were to be prescribed together. The detailed identifications of the mentioned TCM herbal drugs, including Latin names and scientific names, are listed in Supplementary Table S5.

For gastrointestinal diseases, Dahuang, Hsihsin, Fuling, and Banxia were four herbs common in two combinations (Table 5), and even in three combinations (Banxia–Dahuang–Hsihsin) to form herbal pairs with Fuzi. For cardiovascular diseases, Fuzi–Fuling–Baizhu herbal pair had a support value of 5.4% and a lift value of 3.749. Fuzi–Fuling–Baizhu herbal pair was also commonly applied in rheumatological diseases and headaches (Table 5). There was no specific TCM pair for gynecologic diseases gained the support value above 5%. These findings could help Western doctors and researchers to understand the association rules among multiple TCM herbs in specific clinical conditions.

## Discussion

Tseng and Chang proposed that new correlations of TCM herbs could be established by big data analysis, which could trigger further studies from bench to bedside and bring more innovation into clinical practices (Tseng and Chang, 2019). For the first time, we performed AI and data mining methods to generate new knowledge for the modern clinical use of Fuzi and Fuzi-based formulas. Some may argue that Fuzi has become less and less important in modern TCM practice because of its toxicity. However, giving 10% of the study cohort with at least one prescription of Fuzi or Fuzi-based formulas, we insist that Fuzi and Fuzi-based formulas is still an integral and crucial TCM herb. Several new information proposed by the investigation are discussed as follows.

### Modern Clinical Applications of Fuzi and Fuzi-Based Formulas

Contradictory to previous reports tand knowledge, this study showed that the prescriptions of Fuzi were not limited to cardiovascular and rheumatologic diseases, which were emphasized in ancient TCM books. Interestingly, the use of Fuzi in pulmonary, gastrointestinal, and neurologic diseases was more recommended in modern TCM doctors (Figure 2). Our results also revealed that Fuzi and Fuzi-based formulas had modern clinical indications which had never been reported (Table 1). Further, we found that some TCM doctors did not like the fixed ratio of Fuzi-based formulas, but preferred to formulate different proportions of individual TCM herbs. For example, the commercial products of Mahuang–Fuzi–Hsihsin Tang were frequently prescribed for pulmonary diseases (Table 1). Through market basket analysis, Fuzi–Hsihsin–Mahuang was also selected as an important herbal pair with a support value of 9.6% (Lift, 2.379) for pulmonary diseases (Table 5). The results indicate that the proportion of Fuzi, Hsihsin, and Mahuang for pulmonary disease is an important topic worth further studying.

After TCM was integrated into the Western medical system, the combined use of medicine has become an important issue. Understanding the patients’ comorbidities diagnosed by Western doctors also helps TCM doctors to evaluate and prescribe suitable TCM formulas more comprehensively. From this perspective, TCM doctors should be concerned about possible synergic effects and drug–drug interaction while prescribing Fuzi. Our result provided such practical information; for example, long-term Fuzi users frequently suffered from osteoarthritis, peptic ulcers, hypertension, and COPD with the use of NSAIDs, antacids, anti-hypertensive drugs, and COPD inhalants (Table 2 and 3). From another perspective, the results also indicated that some patients might seek TCM as complementary therapy on top of routine Western medical treatments. Therefore, Western doctors also need to understand the relevant precautions of TCM drugs combined with Western medications.

### Rediscover the Practical Fuzi-Based Formulas Hidden in Ancient Traditional Chinese Medicine Books

Market basket analysis was mostly applied to identify concurrent-drug patterns and food consumption. We are first to demonstrate the practicality of market basket analysis in evaluating TCM herbal pairs of more than two drugs. The association rules derived from the market basket analysis lifted the veil of the combination strategies in TCM.

We proposed with scientific data that Fuzi–Dahuang–Hsihsin herbal pair was useful in headache, neurological, and gastrointestinal diseases (Table 5). In a TCM book named Jin Gui Yao Lue (Zhang and Wilms, 2012), which was also called Essential Prescriptions of the Golden Cabinet, initially written by Zhongjing Zhang, we could find this formula to support our analysis. The formula was called Dahuang-Fuzi Tang, a decoction comprised of Dahuang (9 g), processed Fuzi (10 g), and Hsihsin (3 g), prescribed to patients who suffered from abdominal coldness and spasm, migraine, sciatica, and gallbladder and renal stones. However, Dahuang-Fuzi Tang has not become a commercial TCM product and has not been discussed scientifically. We suggest it is a potential botanic drug candidate.

We also founded that Fuzi–Fuling pair was prescribed in many conditions (Table 4). Fuling can expel inappropriate accumulation of water by TCM theory and previous clinical investigation (Huang L. et al., 2018). Thus, Fuzi–Fuling pair may reduce edema, improve heart failure, and enhance the diuretic effect. Through market basket analysis, Fuzi–Fuling pair was frequently combined with Ganjiang, Baizhu, Banxia, or Hsihsin (Table 5). Among these pairs, the Fuzi–Fuling–Ganjiang pair might be simplified and modified from Fuling-Sini Tang used in HF patients or common-cold patients suffering from sweating and irritability. The Fuzi–Fuling–Baizhu pair can be simplified from Jenwu Tang. One commerce product, the Scarlet pill (Zhang and Wilms, 2012), a formula for cold extremities and abdominal pain, in which Wutou (Aconiti Radix) replaced Fuzi, supported the benefit of combinations of Fuzi, Fuling, Banxia, and Hsihsin. These findings enhance the connection between TCM books and current clinical practices and may accelerate the practical commercial Fuzi-based formulas into products.

### The Discovery of Innovative Fuzi-Related Herbal Pairs

The current study discovered some innovative Fuzi-related herbal pairs in specific clinical conditions, which were unknown to most Western researchers. This information, which was extracted from the big database, overcame the knowledge gap in the Western researchers’ minds about using TCM herbs. We not only ignited scientific TCM issues but also proposed issues that need further scientific evidence.

For example, Suanzaoren (Ziziphi Spinosae Semen), which was well known to regulate the central nervous system, improve memory function, depression, and sleep (Li et al., 2013), was found to form a Fuzi–Suanzaoren herbal pair for the neurologic diseases (Table 4). The mechanisms and synergistic effect of Fuzi and Suanzaoren in treating neurologic disease will become a research interest. Another innovative combination that was discovered in this study was Fuzi–Danshen (Salviae Miltiorrhizae Radix et Rhizoma) herbal pair. Danshen was effective in antioxidative, anti-inflammatory, endothelial protective, myocardial protective, anticoagulation, vasodilation, and anti-atherosclerosis mechanisms (Ren et al., 2019). The potentially synergic effect of Fuzi–Danshen herbal pair in cardiovascular diseases has not yet been investigated. Similarly, the cardiovascular effects of Fuzi–Huangqi or Fuzi–Dangqui (Angelicae Sinensis Radix) are also worthy of further research.

Fuzi in combination with Yanhusuo (Corydalis Rhizoma), Chuan-niuxi (Cyathulae Radix), Niuxi (Achyranthis Bidentatae Radix), or Duzhong (Eucommiae Cortex) were frequently prescribed for rheumatologic diseases (Table 4). Among them, Yanhusuo had an antinociceptive effect (Choi et al., 2012). Niuxi and Chuan-niuxi were showed to protect articular chondrocytes (Xu et al., 2017), and Duzhong exhibited anti-osteoarthritic effects (Choi et al., 2020). In TCM theory, these drugs can enhance the effect of Fuzi in treating rheumatologic diseases, but the mechanism was not well understood. The mentioned Fuzi-related herbal pairs can become the next Fuzi-based botanical drugs, as Qili-qiangxin capsules (Tao et al., 2013) and Shenfu injection (Song et al., 2012) were two successful examples derived from Fuzi–related herbal pairs. The current study expanded the research field of Fuzi and the possibility of new drug development.

### Strength and Limitation

The major strength of this study is that it is the first time to evaluate the modern clinical use of Fuzi and Fuzi-based formulas using a large population-based cohort with AI or data mining techniques such as market basket analysis. However, the study results also had some limitations. First, some of the medical records of patients who took TCM decoction but not commercial TCM granular powders were not documented in the Taiwan NHI database. That was because NHI did not pay for TCM decoction in the current TCM system, and patients must pay for themselves. Therefore, the prevalence of Fuzi use might be underestimated. Second, although the Fuzi commercial products were mostly manufactured according to herbal pharmacopeia, the dosage of each commercial powder prescribed by TCM doctors was not standardized and documented in the Taiwan NHI database. Third, we only evaluated the numbers of prescriptions, the prevalence of comorbidities, and concurrent Western medications. The dose-response relationship could not be precisely evaluated. Finally, we evaluated the TCM pairs only with the support values above 5% in the market basket analysis. There might be some interesting TCM pairs with a low prevalence rate that were not discovered.

## Conclusion

The current study is the first to demonstrate the usefulness of market basket analysis in evaluating the combination principle of TCM herbs. We provide epidemiologic evidence on the clinical applications of Fuzi and Fuzi-based formulas in the healthcare system dominated by Western medicine. Our study also supports the traditional usage of Fuzi-based formulas and inspires future pharmacological, observational, and clinical studies of Fuzi-related herbal pairs. In sum, the results light up the road to the development of new Fuzi-based botanic drugs.

## Data Availability

The data analyzed in this study are subject to the following licenses/restrictions: The datasets used and analyzed during the current study are not publicly available, but are available on reasonable request with the permission of the Ministry of Health and Welfare, Taiwan. Requests to access these datasets should be directed to https://dep.mohw.gov.tw/dos/np-2497-113.html.
