# Supplementary material for: Uncovering Modern Clinical Applications of Fuzi and Fuzi-Based Formulas: A Nationwide Descriptive Study With Market Basket Analysis
Source: Front Pharmacol. 2021 Apr 27;12:641530. doi: 10.3389/fphar.2021.641530 (PMC8110898; doi:10.3389/fphar.2021.641530)
Supplement: Supplementary file 1 [file DataSheet1.docx]

| **Supplementary Table 1.** Taiwan National Health Insurance drug codes for Fuzi and Fuzi-based Formulas | |
| --- | --- |
| Items | Specific drug codes |
| Fuzi (附子) | A010565, A014297, A032677, A033813, A043372, A043649, A044520, A045479, A047576, A055829, A056275, A056349, A056369, A056541, A057394, A057471, A057665, A059508 |
| Sini Tang (四逆湯) | A060002, A006039, A009834, A011508, A012097, A013610, A014076, A015782, A016549, A017213, A017215, A017665, A019365, A028434, A028931, A029404, A030300, A031439, A031625, A031903, A032546, A033047, A033131, A034599, A035380, A035927, A037280, A037371, A037459, A037612, A038069, A039226, A041414, A042160, A044410, A044933, A045023, A045217, A045935, A047491, A048580, A048827, A052379, A055216, A056125, A056206, A056920, A059337 |
| Jenwu Tang (真武湯) | A010000, A011536, A013242, A015491, A016619, A016643, A028736, A031469, A031654, A032031, A032993, A033297, A034180, A037767, A038535, A039079, A042788, A045218, A045987, A046111, A046126, A046474, A047393, A056187, A057168, A059344 |
| Mahuang-Fuzi-Hsihsin Tang  (麻黃附子細辛湯) | A012044, A015709, A017261, A017262, A017944, A026310, A026326, A030197, A037001, A037367, A037890, A040634, A040743, A042447, A042678, A044935, A046124, A047245, A047363, A047748, A048615, A055379, A056138, A057334, A059557 |
| Fuzi-Lizhong Tang  (附子理中湯) | A013149, A037905, A038006, A038688, A040875, A042309, A042835, A045716, A046034, A047746, A048916, A052418, A055067, A055363, A056411, A056499, A056838 |

| **Supplementary Table 2.** Systematic diagnosis categories and the corresponding ICD-9-CM codes | |
| --- | --- |
| Category | ICD-9-CM code |
| Headache | 784.xx |
| Neurology | 320.xx–359.xx, 780.xx |
| Psychology | 290.xx–319.xx |
| Sensory organs | 360.xx–389.xx |
| Cardiovascular system | 390.xx–459.xx, 785.xx |
| Pulmonary system | 460.xx–519.xx, 786.xx |
| Gastrointestinal system | 520.xx–579.xx, 787.xx, 789.xx |
| Rheumatology | 710.xx–739.xx |
| Musculoskeletal system | 840.xx–848.xx, 920.xx–924.xx |
| Endocrine system | 240.xx–279.xx, 783.xx |
| Dermatology | 680.xx–709.xx, 782.xx |
| Urology | 580.xx–589.xx, 788.xx |
| Gynecology | 610.xx–679.xx |
| Male reproductive system | 600.xx-608.xx |
| Hematology | 280.xx–289.xx |
| Infectious diseases | 001.xx–139.xx |
| Cancer | 140.xx–208.xx |
| Benign tumors | 209.xx–239.xx |
| Unspecific complaints | Other unspecific ICD-9-CM codes |
| ICD-9-CM = International Classification of Diseases Revision, Ninth Revision, Clinical Modification. | |

| **Supplementary Table 3.** Specific diseases and the corresponding ICD-9-CM codes | |
| --- | --- |
| Disease | ICD-9-CM code |
| Hypertension | 401.xx–405.xx |
| Diabetes mellitus | 250.xx |
| Cerebrovascular disease | 430.xx–438.xx |
| Heart failure | 428.xx |
| Ischemic heart disease | 410.xx–413.xx |
| Cardiac arrhythmias | 427.xx |
| Hyperlipidemia | 272.xx |
| COPD | 491.xx–496.xx |
| Chronic kidney disease | 585.xx |
| Chronic liver disease | 571.xx |
| Esophageal disease | 530.xx |
| Peptic ulcers | 531.xx–534.xx |
| Thyroid disease | 240.xx–246.xx |
| RA, SLE, or AS | 710.xx, 714.xx, 720.xx |
| Osteoarthritis | 712.xx, 713.xx, 715.xx, 716.xx, 720.xx |
| Cancer | 140.xx–208.xx |
| AS = ankylosing spondylitis; COPD = chronic obstructive pulmonary disease; ICD-9-CM = International Classification of Diseases Revision, Ninth Revision, Clinical Modification; RA = rheumatoid arthritis; SLE = systemic lupus erythematosus | |

| **Supplementary Table 4.** Drug classifications and the corresponding ATC codes | |
| --- | --- |
| Drug items | ATC code |
| Aspirin | B01AC06 |
| Clopidogrel | B01AC04 |
| Lipid-lowering agent | *Statins:* C10AA01, C10AA02, C10AA03, C10AA04, C10AA05, C10AA07, C10AA08; *Fibrates:* C10AB01, C10AB02, C10AB03, C10AB04, C10AB05, C10AB06, C10AB09; *Other lipid lowering agents:* C10AC01, C10AC02, C10AC03, C10AD01, C10AD02, C10AD03, C10AD06, C10AD91, C10AX02, C10AX09, C10AX13, C10BA01, C10BA02, C10BA03, C10BA05, C10BX03 |
| Xanthine oxidase inhibitor | M04AA01, M04AA03 |
| Uricosuric agent | M04AB02, M04AB03 |
| Colchicine | M04AC01 |
| ß-blocker | C07AA01, C07AA02, C07AA03, C07AA05, C07AA06, C07AA07, C07AA12, C07AA15, C07AA19, C07AB02, C07AB03, C07AB04, C07AB05, C07AB07, C07AB09, C07AB12, C07AG01, C07AG02 |
| Calcium channel blocker | C08CA01, C08CA02, C08CA03, C08CA04, C08CA05, C08CA06, C08CA07, C08CA08, C08CA09, C08CA12, C08CA13, C08CA15, C08DA01, C08DB01, |
| ACEI/ARB | C09AA01, C09AA02, C09AA03, C09AA04, C09AA05, C09AA06, C09AA07, C09AA08, C09AA09, C09AA16, C09CA01, C09CA02, C09CA03, C09CA04, C09CA06, C09CA07, C09CA08, C09CA09, C09DX01, C09DX03, C09DX04 |
| H2-receptor antagonist | A02BA01, A02BA02, A02BA03, A02BA04, A02BA06 |
| Proton pump inhibitor | A02BC01, A02BC02, A02BC03, A02BC04, A02BC05, A02BC06 |
| NSAID | M01xxxx |
| Metformin | A10BA02 |
| Sulfonylurea | A10BBxx |
| Insulin | A10Axxx |
| COPD inhalant | R03Axxx, R03Bxxx |
| thyroxine | H03AAxx |
| α-adrenoreceptor antagonist | G04CAxx |
| ACEI = angiotensin- converting enzyme inhibitors; ARB= angiotensin receptor blockers; ATC = Anatomical Therapeutic Chemical; NSAIDs = nonsteroidal anti-inflammatory drugs | |

| **Supplementary Table 5.** The detailed identifications of the mentioned herbal drugs, including Latin names and scientific names | | |
| --- | --- | --- |
| **Chinese name** | **Latin name** | **Scientific name** |
| Baizhu (白朮) | Atractylodis Macrocephalae Rhizoma | Dried rhizome of *Atractylodes macrocephala* Koidz |
| Banxia (半夏) | Pinelliae Rhizoma | Dried rhizome of *Pinellia ternata* (Thunb.) Breit |
| Chishao (赤勺) | Paeoniae Rubra Radix | Dried root of *Paeonia veitchii* Lynch or *Paeonia lactiflora* Pallas |
| Chuan-niuxi (川牛膝) | Cyathulae Radix | Dried root of *Cyathula officinalis* Kuan |
| Chuanxiong (川芎) | Chuanxiong Rhizoma | Dried rhizome of *Ligusticum chuanxiong* Hort |
| Dahuang (大黃) | Rhei Radix et Rhizoma | Dried root and rhizome of *Rheum palmatum* L., *Rheum tanguticum* Maxim. ex Balf. or *Rheum officinale* Baill. |
| Danshen (丹蔘) | Salviae Miltiorrhizae Radix et Rhizoma | Dried root and rhizome of *Salvia miltiorrhiza* Bge. |
| Dangqui (當歸) | Angelicae Sinensis Radix | Dried root of *Angelica sinensis* (Oliv.) Diels |
| Duzhong (杜仲) | Eucommiae Cortex | Dried bark of *Eucommia ulmoides* Oliv. |
| Fuling (茯苓) | Poria | Dried sclerotium of *Poria cocos* |
| Fuzi (附子) | Aconiti Lateralis Radix Praeparata | Dried lateral root of *Aconitum carmichaelii* Debeaux |
| Gancao (甘草) | Glycyrrhizae Radix et Rhizoma | Dried root and rhizome of *Glycyrrhiza uralensis* Fisch. |
| Ganjiang (乾薑) | Zingiberis Rhizoma | Dried rhizome of *Zingiber officinale* (Willd.) Rosc. |
| Gegen (葛根) | Puerariae Lobatae Radix | Dried root of *Pueraria lobata* (Willd.) Ohwi |
| Ginseng (人蔘) | Ginseng Radix | Dried root of *Panax ginseng* C. A. Meyer |
| Hsihsin (細辛) | Asari Radix et Rhizoma | Dried root and rhizome of *Asarum heterotropoides* Fr. Schmidt var. *mandshuricum* (Maxim.) Kitag., *Asarum sieboldii* Miq. or *Asarum sieboldii* Miq.var. *seoulense* Nakai |
| Huangqi (黃耆) | Astragali Radix | Dried root of *Astragalus membranaceus* (Fisch.) Bge. var. *mongholicus* (Bge.) Hsiao or *Astragalus membranaceus* (Fisch.) |
| Jiegeng (桔梗) | Platycodonis Radix | Dried root of *Platycodon grandiflorum* (Jacq.) A. DC. |
| Mahuang (麻黃) | Ephedra Herba | Dried stem of *Ephedra sinica* Stapf, *Ephedra equisetina* Bge. or *Ephedra intermedia* Schrenk et C. A. Mey. |
| Niuxi (牛膝) | Achyranthis Bidentatae Radix | Dried root of *Achyranthes bidentata* Bl. |
| Quizhi (桂枝) | Cinnamomi Ramulus | Dried twigs of *Cinnamomum cassia* Presl |
| Rouqui (肉桂) | Cinnamomi Cortex | Dried bark of *Cinnamomum cassia* Presl |
| Sharen (砂仁) | Amomi Fructus | Dried ripe fruit of *Amomum villosum* Lour., *Amomum villosum* Lour. var. *xanthioides* T. L. Wu et Senjen or *Amomum longiligulare* T. L. Wu |
| Suanzaoren (酸棗仁) | Ziziphi Spinosae Semen | Dried and ripe seeds of *Ziziphus jujuba* Mill. var. *spinosa* (Bunge) Hu ex H. F. Chou |
| Wutou (烏頭) | Aconiti Radix | Dried root of *Aconitum carmichaelii* Debeaux |
| Yanhusuo (延胡索) | Corydalis Rhizoma | Dried rhizome of *Corydalis yanhusuo* W.T. Wang |
